# Overexpression of Class III Beta Tubulin and Amplified HER2 Gene Predict Good Response to Paclitaxel and Trastuzumab Therapy

**DOI:** 10.1371/journal.pone.0045127

**Published:** 2012-09-20

**Authors:** Minkyu Jung, Ja Seung Koo, Young Wha Moon, Byeong-Woo Park, Seung Il Kim, Seho Park, Soo Hyun Lee, Soojung Hong, Sun Young Rha, Hyun Cheol Chung, Joo Hang Kim, Joohyuk Sohn

**Affiliations:** 1 Division of Medical Oncology, Department of Internal Medicine, Yonsei Cancer Center, Yonsei University College of Medicine, Seoul, Korea; 2 Department of Pathology, Yonsei University College of Medicine, Seoul, Korea; 3 Department of Surgery, Yonsei University College of Medicine, Seoul, Korea; Innsbruck Medical University, Austria

## Abstract

Through this study, we aimed to validate several biomarkers that have been known to possibly predict the outcomes of the trastuzumab and paclitaxel (TP). Human epidermal growth factor 2 (HER2) positive metastatic breast cancer (MBC) patients who had been treated with TP in single institute from 2006 to 2009 were included in this study. For procured formalin fixed paraffin embedded tumor tissues, HER2 amplification index (AI) and polymorphisms of the immunoglobulin G fragment C receptors (FCGR) were assessed as biomarkers to the trastuzumab and expression of class III beta tubulin (bTubIII) was evaluated as a predictive factor to the paclitaxel. Of 46 patients treated with TP, 27 patients could be evaluated for HER2 AI, 31 for bTubIII, and 26 for FCGR gene polymorphism. The median of the HER2 AI was 5.0 (range, 1.4−15.5) and a higher HER2 AI (≥5.0) was significantly correlated with better response rate (RR) (80% vs. 42%, P = 0.049) and longer progression-free survival (PFS) (13.6 vs. 6.9 months, P = 0.023). High bTubIII expression showed higher RRs than did low expression (81% vs. 40%, P = 0.040) in addition to longer PFS (16.2 months vs. 8.8 months, P = 0.04). However, polymorphisms in FCGR 2A-H131R or FCGR 3A-V158F were not predictive of RR or PFS. Our results suggest that a high HER2 AI and high bTubIII expression could be predictive of the outcomes to TP therapy but no evidence was found in terms of FCGR polymorphisms.

## Introduction

Breast cancer is the most common cancer in women worldwide and the second most common cause of cancer death in women [1]. Although fewer than 10% of women present with metastatic disease at the time of diagnosis, the majority of women who relapse after definitive therapy have disseminated disease rather than an isolated local recurrence [2].

Human epidermal growth factor 2 (HER2) amplified in 20 to 25% of breast cancers is associated with a poor clinical outcome [3]. Trastuzumab, a humanized monoclonal antibody directed against the extracellular domain of HER2 is used either as a single agent or in combination with conventional chemotherapy in metastatic breast cancer (MBC) [4], [5]. Because trastuzumab shows synergistic cardiotoxicity when it is combined with anthracyclines, taxane with trastuzuamab combination therapy is considered a first-line option in metastatic breast cancer [6]. However, the response rate of trastuzumab and paclitaxel (TP) combination therapy is around 36−41% [5], [7], which means the identification of predictive markers for clinical efficacy is an important issue in the management of HER2 positive MBC.

The mechanism of the antitumor activity of trastuzumab is referred to be not only direct anti-proliferative effects [8], but effects on the immune system, including antibody-dependent cell-mediated cytotoxicity (ADCC) and complement-mediated cytotoxicity [9]. In ADCC, trastuzumab binds to tumor cells and is engaged by effecter cells via their immunoglobulin G receptor. Musolino *et al*. reported that the efficacy of trastuzuamab is associated with immunoglobulin G fragment C receptor (FCGR) polymorphisms [10]. Also, there were reported that the magnitude of *HER2/neu* amplification might be a predictive marker for the use of trastuzuamab [11], [12]. In terms of paclitaxel, beta tubulin and p-glycoprotein expression were suggested to be associated with response to paclitaxel [13], [14]. Especially, the expression of class III beta tubulin (bTubIII) was reported to be associated with response to taxane in many cancer types such as non-small cell lung, breast, ovarian, and gastric cancers [13].

With this background, we aimed to address possible predictive biomarkers for HER2-positive metastatic breast cancer patients treated with trastuzuamab and paclitaxel.

## Results

### Patient Characteristics

The clinicopathologic features of 46 patients treated with trastuzumab and paclitaxel are listed in Table 1. All the enrolled patients were HER2 positive according to either immunohistochemistry (IHC) or the fluorescence in situ hybridization (FISH) criteria. The median age was 53 years (range, 33−69 years). The median HER2 AI was 5 (range, 1.4−15.5). Prior (neo) adjuvant chemotherapy was performed in 21 patients (40%) and the remaining 25 patients were chemotherapy naïve metastatic disease from the initial presentation.

**Table 1:** Characteristics of the patients.

Characteristics	Patients	
	No	%
No. of patients	46	100
Median age, years	53	
Range	33−69	
Menopause status		
Pre-	15	32.6
Post	31	67.4
Performance status		
0	20	43.5
1	22	47.8
2	4	8.7
Estrogen receptor status		
Positive	24	52.1
Negative	22	47.9
Progesterone receptor status		
Positive	26	56.5
Negative	20	43.5
HER2 status (IHC)		
2+	5	10.9
3+	41	89.1
HER2 status (FISH)		
Amplification index, median	5	
Range	1.4−15.5	
Disease status at diagnosis		
Relapsed	26	56.5
Initial metastatic	20	43.5
Metastasis site		
Chest wall + regional lymph nodes	34	73.9
Lung	27	58.7
Liver	13	28.3
Bone	28	60.9
Brain	5	10.9
Prior therapy for breast cancer		
Neo/adjuvant chemotherapy	17	40
Anthracyclines	9	52.9
Taxane	6	35.3
Adjuvant hormone therapy	5	10.9
Adjuvant radiotherapy	10	21.7

HER2, Human epidermal growth factor 2; IHC, Immunohistochemistry; FISH, Fluorescent in situ hybridization.

### Clinical Outcomes

**Figure 1:**
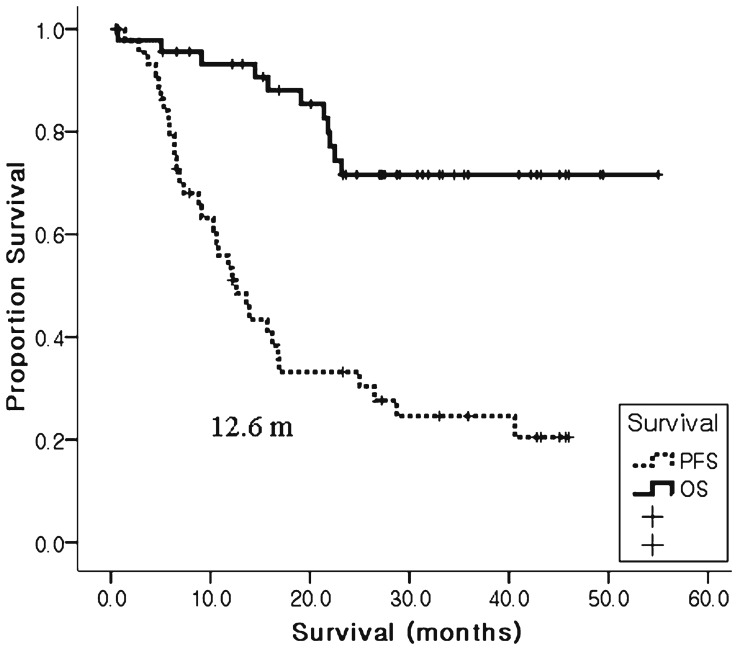
Kaplan-Meier estimates of median progression-free survival (PFS, dotted line) and overall survival (OS, straight line) for all patients (N = 46). The median survival of PFS is 12.6 months and the median OS was not yet reached.

Forty patients (87%) have completed six cycles of paclitaxel and trastuzumab and continued on following trastuzumab maintenance therapy. Response evaluation was performed in 44 patients. Two patients were missed because one died from pneumonia followed by septic shock after the first cycle of chemotherapy and the other was lost to follow-up after the first cycle of therapy. In the remaining 44 patients, the overall response rate was 63.6%, including complete response (CR) in five (11.3%) and partial response in 23 (52.3%) (Table 2). Two patients with cervical neck node metastasis achieved CR after six cycles of the trastuzumab and paclitaxel (TP), and then one patient received radiotherapy on neck node and the other underwent neck node dissection and finally reported pathologic CR. Two patients who had liver and lung metastasis showed radiologic CR after six courses of TP and one patients developed controlateral breast metastasis after 24 months of trastuzuamab treatment and one patient was lost to follow-up after 9 months of treatment. The last patient who had metastasis in the brain alone underwent radiosurgery and CR has been observed for 14 months. The median progression-free survival (PFS) was 12.6 months [95% confidence interval (CI), 7.7−19.5 months] and the median OS was not reached with median follow-up of 23.6 months (Figure 1).

**Table 2:** Response to therapy.

Response	Evaluable patients (n = 44)	
	No.	(%)
Complete response	5	11.3
Partial response	23	52.3
Stable disease	15	34.1
Progressive disease	1	2.3
Overall response rate	28	63.6

### Predictive Factors

**Figure 2:**
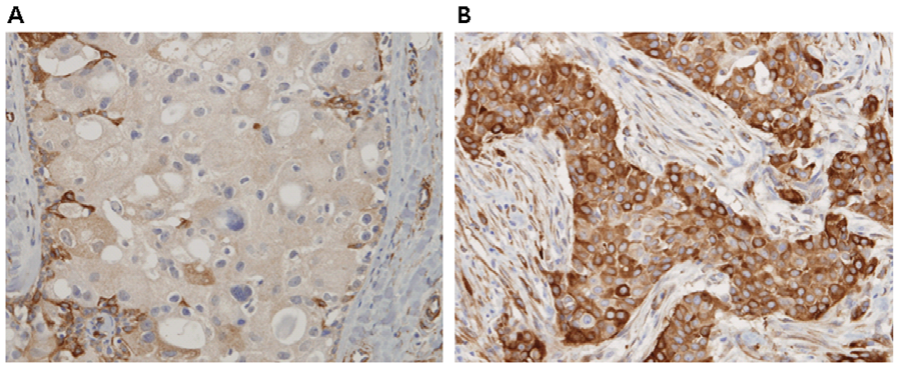
A and B were representative staining for class III beta tubulin in invasive carcinoma of the mammary gland; absence of staining (A), and strong staining(B).

**Figure 3:**
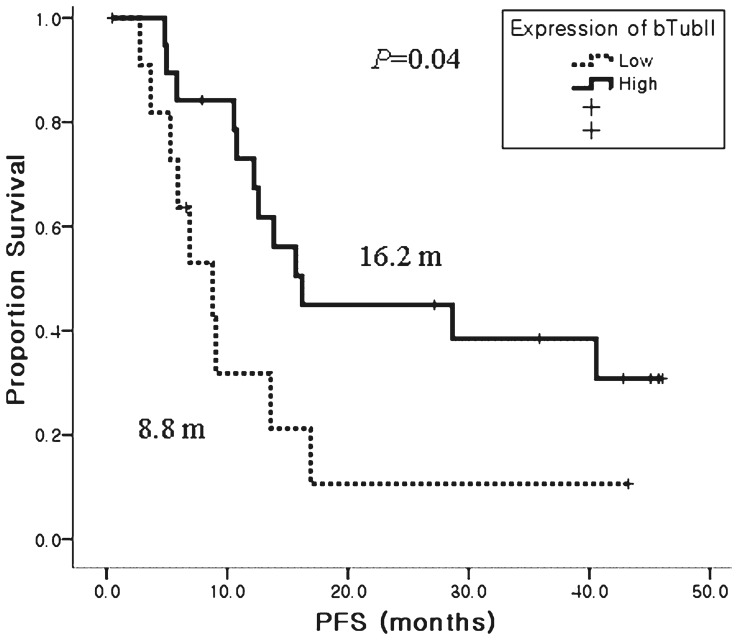
Kaplan-Meier curves comparing PFS according to expression of class III beta tubulin. The patients with high expression of bTubIII tumors had significant longer PFS compared with those with low-expression tumors (16.2 months vs. 8.2 months, p = 0.004).

Table 3 shows response rates (RRs) according to clinicopathologic parameters and markers evaluated as potential predictive factors. Tissue was available for bTubIII expression analysis in 31 patients (67.4%) and the median IHC expression score for bTubIII was 40% (range, 0−90%). At an IHC cutoff score of 40%, bTubIII expression was high in 21 (67.7%) and low in 10 patients (Figure 2). Patients with bTubIII-high tumors showed an 81% response rate compared to 40% in patients with bTubIII-low tumors (P = 0.045). Patients with bTubIII-high tumors also showed a longer PFS than bTubIII-low tumors (16.2 months vs. 8.8 months, P = 0.04) (Figure 3).

**Table 3:** Clinical response according to baseline characteristics and biomarkers.

	Number (%)	Response (%)		P-value
		CR/PR	SD/PD	
Age				0.104
<60	28 (63.6)	15 (53.6)	13 (46.4)	
≥60	16 (36.4)	13 (81.3)	3 (18.8)	
Performance status				0.652
0−1	38 (86.4)	25 (65.8)	13 (34.2)	
2	6 (13.6)	3 (50)	3 (50)	
Hormone receptor status				0.521
Negative	16 (36.4)	12 (75)	4 (25)	
Positive	28 (63.6)	18 (64.3)	10 (35.7)	
No. of metastasic site				0.336
0−1	16 (36.4)	4 (25)	12 (75)	
≥3	28 (63.6)	17 (60.7)	11 (39.3)	
FCGR 2A (n = 26)				
HH	13 (50)	8 (61.5)	5 (38.5)	0.89
HR	12 (46.2)	9 (75)	3 (25)	
RR	1 (3.8)	1 (100)	0	
FCGR 3A (n = 26)				0.927
VV	1 (3.5)	1 (100)	0	
FV	9 (34.6)	6 (66.7)	3 (66.3)	
FF	16 (61.5)	11 (68.7)	5 (31.3)	
FISH ratio (n = 27)				0.049
<5	12 (44.4)	5 (41.7)	7 (58.3)	
≥5	15 (55.6)	12 (80)	3 (20)	
Expression of bTubIII (n = 31)				0.04
Low	10 (32.3)	4 (40)	6 (60)	
High	21 (67.7)	17 (81)	4 (19)	

FCGR, Immunoglobulin G fragment C receptors; FISH, Fluorescent in situ hybridization. CR, complete response; PR, partial response; SD, stable diseases; PD, progression of disease.

**Figure 4:**
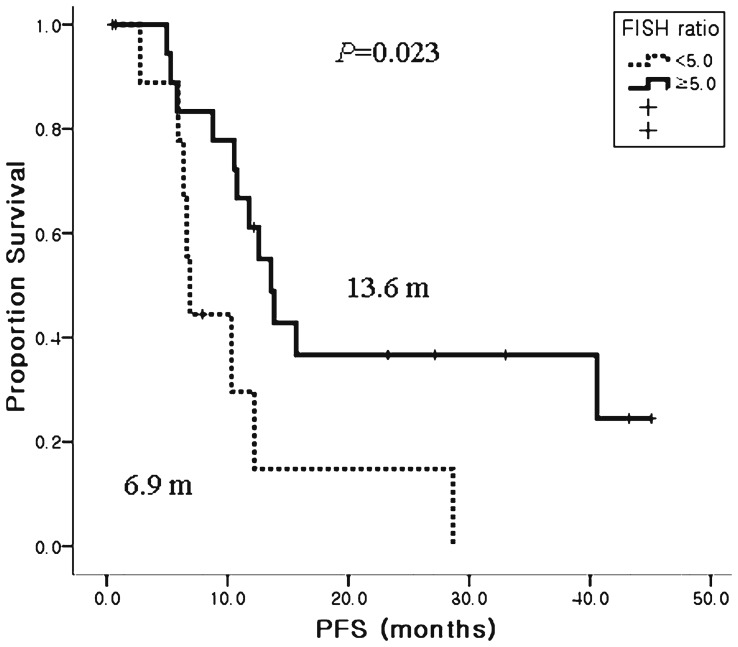
Kaplan-Meier estimates of PFS according to HER2 FISH amplification index (AI). The patients with high HER2 FISH AI tumors had significant.

In the 27 patients for whom HER2 FISH data were available, HER2 AI varied from 1.4 to 15.4, and the median AI was 5. Patients with a low HER2 AI (<5) had a lower response rate (RR) (41.7% vs. 80%, P = 0.049) and a significantly shorter survival than high HER2 AI group (6.9 months vs. 13.6 months, P = 0.023) (Figure 4).

FCGR polymorphisms were evaluated in 26 patients. The frequencies of FCGR 2A-H131R genotypes were H/H 50%, H/R 46.2%, and R/R 3.8%. For FCGR 3A-V158F, frequencies were V/V 3.8%, V/F 34.6%, and F/F 61.6%. However, no differences in either RR or PFS according to FCGR polymorphism were observed.

Good performance status [Eastern Cooperative Oncology Group (ECOG) 0 or 1], high expression of bTubIII, and high HER2 AI were significantly associated with PFS. However, they were not observed to be independent predictive markers for PFS by multivariate analysis (Table 4).

**Table 4:** Univariate and multivariate analysis for progression free survival.

	Univariate analysis		Multivariate analysis	
	Median months (95% CI)	P-value	Hazard ratio (95% CI)	P-value
Age		0.064		
<60	12.2 (8.7−15.7)			
≥60	15.7 (7.4−23.9)			
Performance status		0.01		0.18
0−1	13.6 (8.6−18.6)		1	
2	5.8 (4.6−7.1)		2.45 (0.66−9.51)	
Hormone receptor status		0.392		
Negative	15.7 (11.4−19.9)			
Positive	10.8 (8.3−13.2)			
No. of metastasic site		0.14		
0−1	13.9 (9.2−18.5)			
≥3	10.8 (4.6−17.0)			
FCGR 2A		0.836		
HH	16.2 (13.8−18.6)			
HR/RR	25.0 (2.2−47.9)			
FCGR 3A		0.537		
VV/FV	11.8 (9.1−14.5)			
FF	25.0 (10.8−39.2)			
Expression of bTubIII		0.04		0.164
Low	8.8 (4.6−13.0)		1	
High	16.2 (11.4−21.0)		0.47 (0.17−1.36)	
FISH ratio		0.023		0.184
<5	6.9 (6.1−7.7)		1	
≥5	13.6 (11.1−16.0)		0.46 (0.15−1.44)	

CI, Confidence interval; FCGR, Immunoglobulin G fragment C receptors; FISH, Fluorescent in situ hybridization.

### Toxicity

In general, treatments were well tolerated and the majority of adverse events were of mild to moderate severity (Table 5). One patient experienced treatment-related death from pneumonia with septic shock after receiving the first cycle of chemotherapy, and G3 or 4 neutropenia was seen in 11 patients (23.9%). The most common nonhematologic toxicity was peripheral neuropathy and paclitaxel was discontinued in one patient for grade 3 neuropathy after four treatment cycles. There were no cases of grade 3 or 4 cardiac dysfunction, while 8 of the 46 patients (16.5%) developed asymptomatic grade 1 or 2 left ventricle dysfunction.

**Table 5:** Hematological and non-hematological toxicities.

Toxicity	Grade 1		Grade 2		Grade 3		Grade 4	
	No.	%	No.	%	No.	%	No.	%
Hematological								
Neutropenia	7	15.2	9	19.6	6	13	5	10.9
Anemia	10	21.7	5	10.9	3	6.5	0	0
Thrombocytopenia	2	4.3	2	4.3	1	2.2	0	0
Non-hematological								
Nausea and vomiting	5	10.9	2	4.3	0	0	0	0
Neuropathy	10	10.9	11	23.4	1	2.1	0	0
LV dysfunction	5	10.9	3	6.5	0	0	0	0

LV, Left ventricle.

## Discussion

The aim of this study was to validate predictive markers for the efficacy of paclitaxel and trastuzuamab in recurrent or metastatic breast cancer patients. We found that overexpression of bTubIII and amplified HER2 genes predicted good response and favorable progression free survival in HER2-positive breast cancer patients treated with paclitaxel and trastuzuamab.

Preclinical studies have reported that overexpression of bTubIII is associated with taxane resistance in many cancer cell lines [15]–[18]. In a clinical setting, high levels of bTubIII expression predict lower response and poor outcomes in non-small cell lung cancer, ovarian cancer, breast cancer, and primary unknown-site cancer [19]–[22]. However, we found the contrary results, with patients with high bTubIII expression having a higher RR and longer PFS than those with low bTubIII expression. This results are consistent with some other studies reporting that overexpression of bTubIII is associated with good response and favorable prognosis in ovarian cancer, non-small cell cancer and breast cancer [23]–[25]. The reason for this discrepancy is not yet known. However, potential explanations include that currently validated predictive markers are mixed predictive and prognostic factors [26]. According to Reinman *et al*., high bTubIII expression is a poor prognostic factor for non-small cell lung cancer patients treated with surgery alone, although the benefit of adjuvant chemotherapy was greater in patients with high bTubIII expression [24]. This is similar to the story of HER2 in breast cancer, where HER2 is a poor prognostic marker but a good predictive marker to trastuzumab [27]. A second explanation is that studies were different each other in terms of cut-off levels for bTubIII positivity and chemotherapy agents combined with taxane. The third explanation is differential predictive implications in early [24] versus advanced settings [19] or in serous cell-type ovarian carcinoma [20] versus clear cell ovarian carcinoma [23]. Another possible cause of discrepancies is the limitations of each study, such as retrospective design, small sample size, or heterogeneity of the eligible patients (with different numbers of previous lines of treatment).

The influence of FCGR genotype on treatment outcome has been reported in breast cancer [10] and other malignancies [28]–[30]. According to the report from Dr. Musolino, a significant difference in RR was observed between patients with FCGR 3A V/V and those with either 158 V/F or 158 F/F genotype (82% v 42% v 35%; P = .03) [10]. However, we found no difference in RR or survival according to FCGR polymorphism in our study. Fifty-six patients would be needed to detect a difference in RR based on genotypes of FCGR (in the Musolino’s study) at a power of 80% and p-value of 0.05 using Fisher’s exact test. Accordingly, our study did not meet the statistical power, because genotypes of FCGF were performed in only 26 patients. However, the genotypes frequencies and clinical responses according to genotypes of FGFR were quite different between this study and Musolino’s study (Supplementary table S1). In our study, VV type of FCGR 2A was identified in only one patient (3.5%), which was a much lower incidence than Musolino’s data (20.4%), and the RR according to FV and FF genotypes were quite similar with 66.7% and 68.7%, respectively (p = 0.927) (Supplementary table S1). Therefore, even if the sample size in our study was increased, a difference of RR based on FCGR 3A genotypes might not be observed. Like the this study, the recently reported largest analysis to evaluate the association between FCGR polymorphisms and trastuzuamab efficacy in HER-2-positive breast cancer failed to confirm results from previously published smaller studies [31].

Regardless of strong points that this study was performed in quite homogenous group of patients, that is, HER2 positive MBC breast cancer patients who received TP as the first line therapy and that all the test was performed prospectively with the help of the independent pathologist, it has several limitations. First, we had a small sample size and the three markers (bTubIII, HER2 FISH ratio, and FCGR polymorphism) were evaluated in only part of the patients because of limited available tumors and blood samples. Second, our follow-up duration was insufficient. At a median follow-up of 23.6 months, 31 of 46 patients (67.4%) experienced disease progression and only 11 deaths (23.9%) had occurred. Therefore, the association between several factors and OS could not be evaluated. Consequently, these factors should be validated in large populations with long-term follow-up.

In conclusion, we found that high bTubIII expression and high HER2 AI are good predictive markers for RR and favorable prognosis in HER2-positive metastatic breast cancer patients treated with paclitaxel and trastuzuamab. However, no evidence was found supporting an effect of FCGR polymorphisms on therapeutic benefit of the trastuzuamab. Large scaled prospective studies are needed to confirm the roles of these biomarkers in HER2-positive breast cancer patients treated with TP.

## Methods

### Patients

We recruited 46 women with HER2 positive metastatic or recurrent breast cancer. All of the patients underwent biopsy of a primary or recurred breast tumor before the first-line chemotherapy with trastuzumab and paclitaxel at the Yonsei University Health System between September 2006 and October 2009.

Paclitaxel (175 mg/m^2^ i.v., every three weeks) and trastuzumab (4 mg/kg i.v., as a loading dose, then 2 mg/kg weekly) were administered to patients with HER-2-positive metastatic breast cancer as the first-line chemotherapy. Six cycles of combination therapy with paclitaxel and trastuzumab were administered followed by trastuzumab until disease progression. The study was approved by the institutional review board. Tumor assessments were performed every two cycles, and disease response was categorized as complete response (CR), partial response (PR), stable disease (SD), and progressive disease (PD) according to the response criteria in solid tumor (RECIST, version 1.1) criteria [32]. Toxicity was scored every 3 weeks according to the Common Toxicity Criteria of the National Cancer Institute (NCI-CTC, version 4.1) [33].

### HER2 Positivity and Amplification Index

Serial 4-µm sections of tissue were used for both IHC and FISH test for HER2. IHC was performed using a Hercep Test Kit (DAKO, Carpinteria, CA) and results were interpreted semi-quantitatively using scale values of 0, 1+, 2+, and 3+ according to the manufacturer’s recommended scoring system. Scoring was conducted by a pathologist unaware of the patients’ clinical information. FISH was performed using a PathVysin *HER2* DNA probe kit (PathVysin; Vysis, Stuttgart-Faranenhof, Germany) and was analyzed as previously described [34]. The HER2 amplification index (AI) was defined as the number of HER2/neu signals per nucleus relative to the number of chromosome 17 centromere signals per nucleus in FISH. Tumor were considered to be amplified if this ratio was greater than two and amplified tumors were further categorized as lower amplified or higher amplified group with the cut-off value of the median HER2 AI in whole recruited patients. HER2 positivity was defined as an intensity of 3+ according to IHC or as gene amplification in FISH.

### IHC for bTubIII

Expression of bTubIII was evaluated using IHC with a primary antibody against bTubIII (1∶50, Etipomics, 1967-1, USA). To evaluate bTubIII expression, 4-µm tissue sections were deparaffinized in xylene, rehydrated and treated with 2.5% H_2_O_2_ for 10 min to block endogenous peroxidase. Slides were subjected to heat-induced antigen retrieval for 2 min in 10 mmol/L citrate buffer (pH 7). After washing with peripheral blood smear examination (PBS), slides were incubated with primary antibody for 90 min at room temperature. A second PBS wash was followed by incubation at room temperature for 30 min with a mouse secondary antibody and another PBS wash. Color development occurred via incubation with 0.5% 3,3-diaminobenzidine solution for 1 min. After washing with PBS, the slides were stained with Harris hematoxylin and submitted for interpretation. Immunoreactivity for bTubIII was evaluated in three areas per slide for correlation and confirmation of the tissue diagnosis. The number of tumor cells with cytoplasmic staining of bTubIII was counted. Possible scores ranged from 0 to 100%. Cutoffs for definition of “low” or “high” expression of bTubIII by IHC were defined based on the median values observed in the patient population.

### FCGR Polymorphisms

Peripheral blood (10 cc) was obtained from enrolled patients who provided signed informed consent for blood analysis. Genomic DNA was purified from leukocytes after selective lysis of erythrocytes using an automated DNA extractor, according to the manufacturer’s instructions (Applied Biosystems, Foster City, CA). FCGR 2A genotyping was performed on genomic DNA using polymerase chain reaction (PCR) and polymorphism assignment determined by restriction enzyme digestion, followed by agarose gel electrophoresis, using previously described methods [35]. FCGR3A genotyping was performed using allele-specific PCR methods as previously described [36].

### Statistical Methods

Clinical characteristics and response rates were compared based on bTubIII expression, HER2 AI, and FCGR polymorphisms using a χ^2^ test or Fisher’s exact test. Survival estimates were calculated using the Kaplan-Meier method. PFS was calculated from the first date of chemotherapy to the date of disease progression or of death from any cause. Differences in PFS according to expression of bTubIII, HER2 AI, and FCGR polymorphism were compared using the log-rank test. Multivariate analysis used Cox’s proportional hazard model to define independent prognostic factors for hazard ratios, and the 95% CI was estimated for significant values according to univariate analysis. Statistical data were obtained using SPSS software, version 12.0 (SPSS Inc, Chicago, IL).

### Ethics Statement

The study was done in accordance with the Declaration of Helsinki. This study was approved by the Institutional Review Board of Yonsei University Health System (4-2009-0166). We received written informed consent from the patients who were enrolled in this study.

## Supporting Information

Table S1 **Clinical response according to FCGR genotypes between Mussolino’s and current study.**
(DOC)
