# Supplementary material for: Overexpression of Class III Beta Tubulin and Amplified HER2 Gene Predict Good Response to Paclitaxel and Trastuzumab Therapy
Source: PLoS One. 2012 Sep 20;7(9):e45127. doi: 10.1371/journal.pone.0045127 (PMC3447874; doi:10.1371/journal.pone.0045127)
Supplement: Table S1 — Clinical response according to FCGR genotypes between Mussolino’s and current study. (DOC) [file pone.0045127.s001.doc]

**Supplementary table S1.** Clinical response according to FCGR genotypes between Mussolino's and current study

|  |  | Musolino's study (N=54) [10] | | | |  | Current study (N=26) | | | |
| --- | --- | --- | --- | --- | --- | --- | --- | --- | --- | --- |
|  |  | Number (%) | Response (%) | | p-value |  | Number | Response (%) | | p-value |
|  |  |  | CR/PR | SD/PD |  |  |  | CR/PR | SD/PD |  |
| FCGR 2A |  |  |  |  | 0.17 |  |  |  |  | 0.89 |
|  | HH | 10 (18.5) | 7 (70) | 3 (30) |  |  | 13 (50) | 8 (61.5) | 5 (38.5) |  |
|  | HR | 34 (63.0) | 15 (44) | 19 (56) |  |  | 12 (46.2) | 9 (75) | 3 (25) |  |
|  | RR | 10 (18.5) | 4 (40) | 6 (60) |  |  | 1 (3.8) | 1 (100) | 0 |  |
| FCGR 3A |  |  |  |  | 0.03 |  |  |  |  | 0.927 |
|  | VV | 11 (20.4) | 9 (82) | 2 (18) |  |  | 1 (3.5) | 1 (100) | 0 |  |
|  | FV | 26 (48.1) | 11 (42) | 15 (58) |  |  | 9 (34.6) | 6 (66.7) | 3 (33..3) |  |
|  | FF | 17 (31.5) | 6 (35) | 11 (65) |  |  | 16 (61.5) | 11 (68.7) | 5 (31.3) |  |

FCGR, Immunoglobulin G fragment C receptors; CR, complete response; PR, partial response; SD, stable diseases; PD, progression of disease
